# Patient-Specific Cardiac Magnetic Resonance Feature Tracking Approach for Scar Detection in Concomitant Ischemic and Non-Ischemic Heart Disease

**DOI:** 10.26502/fccm.92920297

**Published:** 2022-12-23

**Authors:** Malgorzata Polacin, Tobias Hünermund, Oliver Müggler, Hatem Alkadhi, Sebastian Kozerke, Robert Manka

**Affiliations:** 1 Institute of Diagnostic and Interventional Radiology, University Hospital Zurich, University of Zurich, Raemistrasse 100, 8091 Zurich, Switzerland; 2 Institute for Biomedical Engineering, University and ETH Zurich, Gloriastrasse 35, 8092 Zurich, Switzerland; 3 Department of Cardiology, University Heart Center, University Hospital Zurich, University of Zurich, Raemistrasse 100, 8091 Zurich, Switzerland

**Keywords:** Cardiac Magnetic Resonance, Feature Tracking, Ischemic Heart Disease

## Abstract

**Aim:**

This study investigated a patient-specific approach of using cardiac magnetic resonance (CMR) feature tracking for scar detection in a heterogenous patient group with chronic ischemic and non-ischemic heart disease.

**Methods:**

CMR exams of 89 patients with concomitant chronic ischemic and non-ischemic heart disease (IHD+) as well as 65 patients with ischemic scars only (IHD) were retrospectively evaluated. In all patients, global (GCS) and segmental circumferential strain (SCS) was derived from native cine images using a dedicated software (Segment CMR, Medviso). After calculation of patient-specific median GCS (GCS_median_), segmental values from GCS_median_ percentage plots were correlated with corresponding myocardial segments in late gadolinium enhancement (LGE).

**Results:**

Overall GCS ranged between −3.5% to −19.8% and average GCS was lower in IHD+ than in IHD (p <0.05). In IHD, 19% of all myocardial segments were infarcted, in IHD+ 16.6%. Additionally, non-ischemic LGE was present in 6.7% of segments in IHD+. Correlation of GCS_median_ percentage plots with corresponding LGE showed that presence of ischemic scar tissue in a myocardial segment was very likely below a cut-off of 39.5% GCS_median_ (87.5% sensitivity, 86.3% specificity, AUC 0.907, 95% CI 0.875-0.938, p < 0.05).

**Conclusion:**

In patient-specific GCS_median_ percentage plots calculated from native cine images, ischemic scar tissue can be suspected in myocardial segments below the threshold of 40% GCS_median_ (sensitivity 88%, specificity 86%), even in a heterogenous patient cohort with ischemic and non-ischemic heart disease.

## 1. Introduction

Cardiac magnetic resonance (CMR) late gadolinium enhancement (LGE) became essential for imaging scar tissue in ischemic heart disease [1]. Clinically established native techniques for detection of ischemic scars are still limited with reduced diagnostic power of CMR exams in patients with ischemic heart disease and contraindications to gadolinium. CMR feature tracking measures myocardial deformation and provides information about global and segmental strain derived from routinely acquired native cine sequences by tracking previously registered voxels throughout the cardiac cycle [2-4]. Segmental circumferential strain showed considerable potential of distinguishing scar tissue from remote myocardium based on reduced tissue deformation properties in infarcts compared to strain values of adjacent healthy myocardium [5-8]. However, impairment of strain is not specific for ischemic damage and strain values may be altered by various cardiac conditions like cardiomyopathies or other non-ischemic cardiac diseases [9]. Moreover, defining universally valid thresholds for infarcted and remote myocardium in heterogenous patient groups seems challenging due to inter-individual variability of global strain values. Therefore, this study investigates a patient- specific approach for scar detection using a threshold based on patient-specific median global circumferential strain (GCS_median_) in patients with ischemic and with both ischemic and non- ischemic heart disease.

## 2. Methods

### 2.1 Study Population

**Figure 1:**
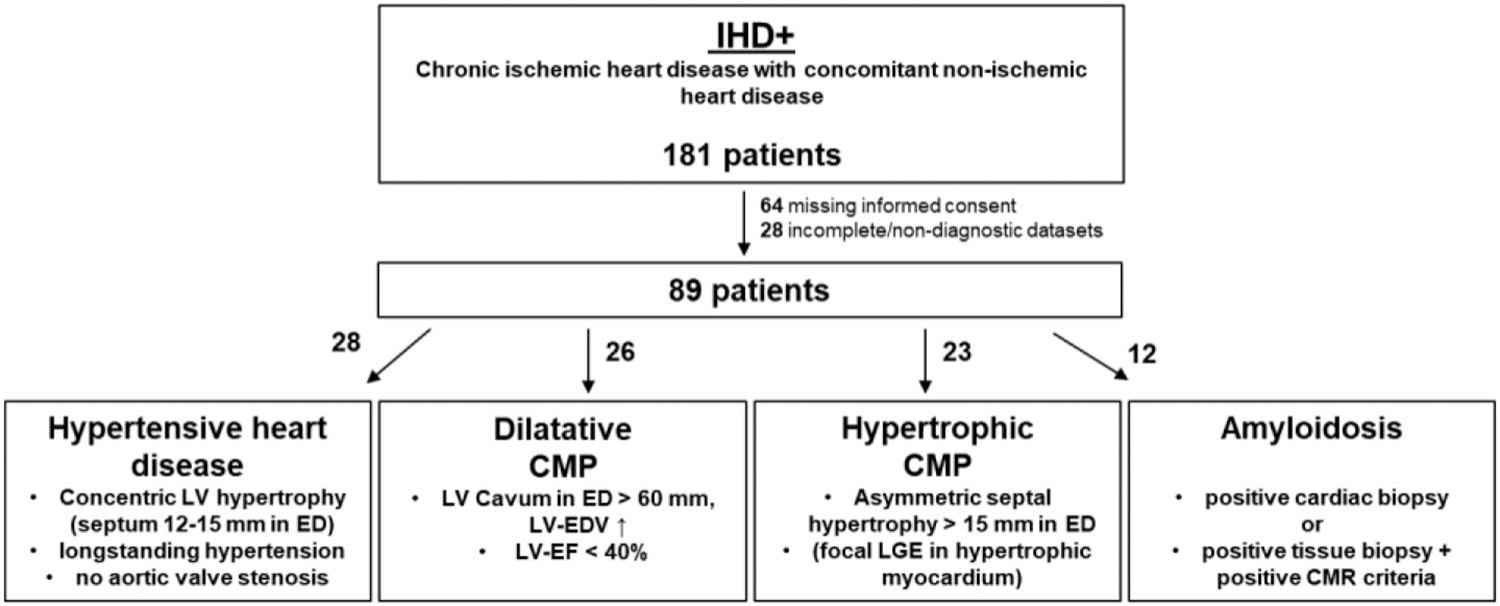
IHD+ group profile. After exclusion of 92 patients, 89 patients with both chronic ischemic and non-ischemic heart disease were enrolled in IHD+ group: 28 patients with hypertensive heart disease, 26 patients with severe left ventricular dilatation (dilatative cardiomyopathy), 23 patients with hypertrophic cardiomyopathy and 12 patients with amyloidosis. CMP: Cardiomyopathy; ED: End-Diastole/End-Diastolic; IHD+: Patients with Concomitant Chronic Ischemic and Non-Ischemic Heart Disease; LGE: Late Gadolinium Enhancement; LV: Left Ventricle/Left-Ventricular; LV-EDV: Left Ventricular End-Diastolic Volume; LV-EF: Left Ventricular Ejection Fraction

This retrospective study had institutional review board and local ethics committee approval and all included patients provided written informed consent. This study analysed patients undergoing CMR exams between September 2017 and January 2020 in our department. From 181 patients with both chronic ischemic and non-ischemic heart disease, 89 patients could be enrolled (“ischemic heart disease+” [IHD+]); Figure 1). Of these 89 patients with ischemic heart disease, 28 patients additionally had diagnosis of hypertensive heart disease, 23 had hypertrophic cardiomyopathy, 26 had severe LV dilatation and 12 patients had proven amyloidosis [10-12]. From the same time interval, 65 patients who were diagnosed with 85 chronic ischemic scars without evidence of other, non-ischemic cardiac conditions (“ischemic heart disease”/IHD) were additionally enrolled (Table 1). Patients with evidence or suspect of an acute cardiac condition (acute chest pain, suspicious electrocardiogram, elevated cardiac enzymes, relevant coronary stenosis in invasive angiography) in the last 6 months before CMR were excluded.

### 2.2 CMR Data Acquisition

All CMR exams were conducted on a clinical 1.5T MR (Achieva, Philips Healthcare). Cine balanced steady-state free precession (bSSFP) images in long-axis geometries (2-, 3- and 4- chamber view) as well as in short axis orientation covering the left ventricle (LV) (field of view: 350 × 350 mm^2^; repetition time/echo time: 3.0/1.5 ms; spatial resolution 1.2 × 1.2 x 8 mm^3^; number of cardiac phases: 50) were acquired for functional LV assessment. After acquisition of cine images, Gadolinium (0.2 mmol gadobutrol [Gadovist; Bayer Schering Pharma, Zurich, Switzerland] per kilogram body weight) was applied for LGE sequences (inversion recovery gradient-echo sequence; field of view: 350 × 350 mm^2^; repetition time/echo time: 3.5/1.7 ms; spatial resolution 1.2 × 1.2 × 8 mm^3^; inversion time: 205–250 ms; flip angle: 15°) in short axis orientation and in 2-,3- and 4 chamber view.

### 2.3 CMR Data Analysis

#### 2.3.1 CMR feature tracking:

**Figure 2:**
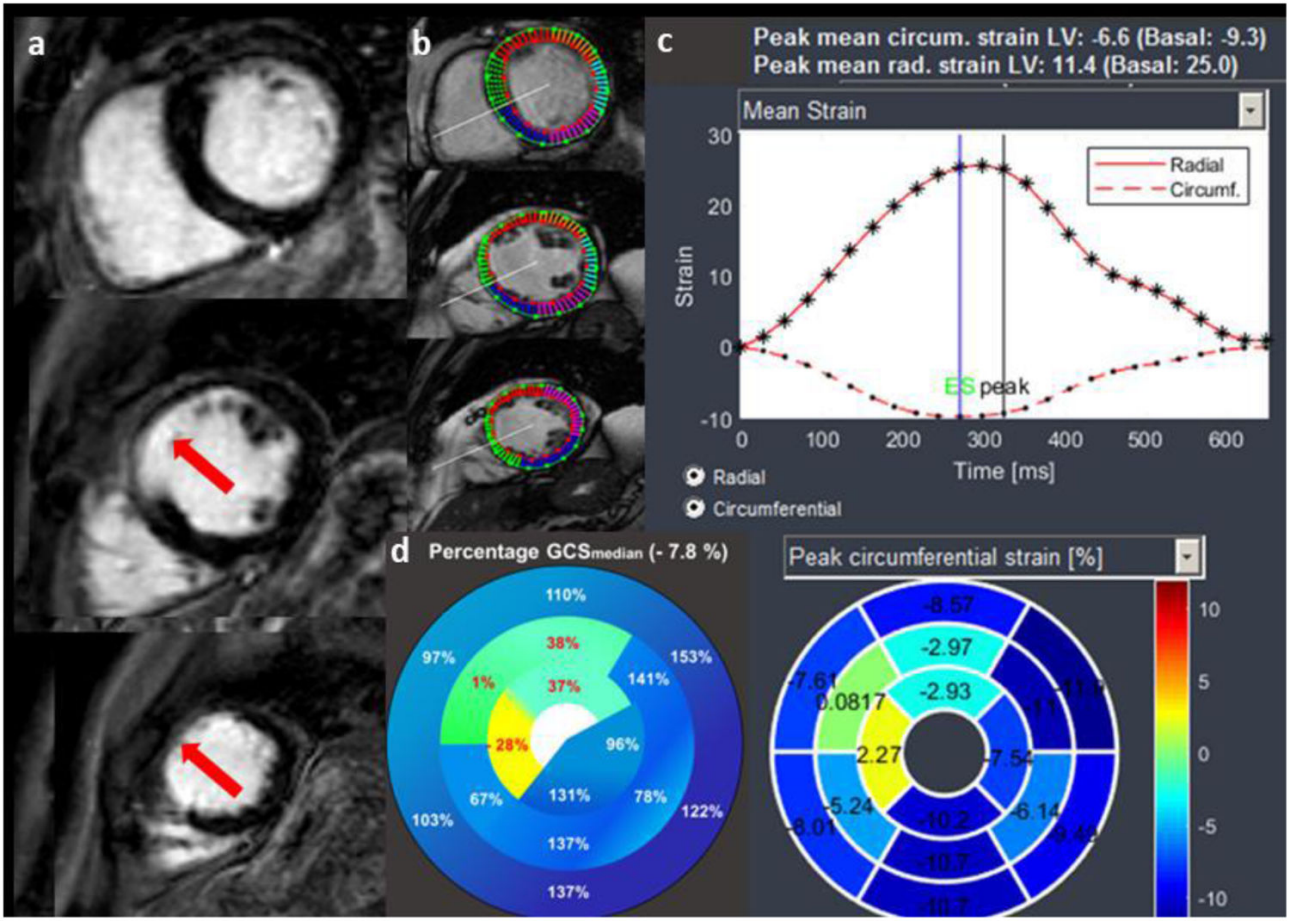
55-year-old male patient with chronic LAD infarction. 2a: Ischemic LGE in AHA segment 7,8,13 and 14 (red arrows) in a 55-year-old patient with chronic ischemic heart disease. 2b: Endo- and epicardial contouring of corresponding cine short axis slices preceding strain calculation (demonstrated by a basal, midventricular and apical slice). 2c: Circumferential strain calculation with a polar plot map depicting segmental values; mean GCS in this patient is −6.6%. 2d: Based on segmental strain values, patient-specific median GCS (GCS_median_) was calculated and individual GCS_median_ percentage polar plot maps were generated. The resulting percentage value of GCS_median_ of every segment was correlated with corresponding myocardial segment in LGE short axis. AHA- American Heart Association; GCS/SCS- Global/Segmental Circumferential Strain; LAD- Left Anterior Descending Coronary Artery; LGE- Late Gadolinium Enhancement

Global (GCS) and segmental (SCS) circumferential strain was derived from native cine short axis stacks (anonymized data) using a specific software (Segment CMR v3.0, Medviso; Figure 2) as previously described [7] with both readers (M.P. and T.H.) being blinded to each other, to patient information and to LGE sequences. In every patient, patient- specific median GCS (GCS_median_) was calculated from the segmental circumferential strain values. Subsequently, percentages of GCS_median_ were visualized in an individualized polar plot map (Figure 2d) and segmental values were correlated with corresponding myocardial segments in LGE short axis (reference standard) [13].

#### 2.3.2 Assessment of LV Function and LGE Images:

For the radiological report, ventricular volumes and function were calculated using IntelliSpace Portal (Philips, Version 8.0.3). LGE was classified as “ischemic LGE” (subendocardial or transmural LGE with accompanying regional wall motion abnormality) or “non-ischemic LGE” (LGE in a midmyocardial, epicardial or circular subendocardial/diffuse distribution without concomitant wall motion pathology). In ischemic LGE, segments with infarct transmurality above 50% wall thickness were considered “non-viable” [14]. Every CMR report was revised by a EACVI level III cardiologist with > 20 years of experience in CMR (R.M.).

### 2.4 Statistical Analyses

Statistical analyses were conducted using commercially available software (IBM SPSS Statistics, release 25.0; SPSS, Armonk, NY). Categorical variables were expressed as frequencies or percentages, continuous variables were expressed as means ± standard deviations. Global and segmental circumferential strain values were compared using two-tailed paired *t*-tests or Wilcoxon signed rank tests. To measure interobserver agreement, the intraclass correlation coefficient (ICC) was used; ICC = 0.50- 0.75 was considered moderate, ICC = 0.75- 0.9 was considered good and ICC > 0.9 was considered excellent agreement [15]. Receiver operating characteristics (ROC) were calculated and the area under the curve (AUC) was determined for the percentage of GCS_median_ to differentiate infarcted from remote myocardium; optimal cut-off was defined by the Youden’s index. A two-sided p-value < 0.05 indicated statistical significance.

## 3. Results

### 3.1 Late Gadolinium Enhancement in IHD and IHD+

#### 3.1.1 Ischemic Heart Disease (IHD):

In IHD, 210 out of 1105 segments (19%) had ischemic LGE; 194 of 210 infarcted segments (92.4%) were non-viable.

#### 3.1.2 Concomitant Ischemic and Non-Ischemic Heart Disease (IHD+):

In IHD+, 251 out of 1513 (16.6%) segments had ischemic LGE with mostly non-viable infarcts (94%, 236 out of 251). Furthermore, 101 segments (6.7%) showed non-ischemic LGE. In patients with hypertensive heart disease, 3 segments showed midmyocardial LGE antero-/inferoseptal basal and 4 segments showed epicardial LGE at the basal RV junction. In patients with dilatative cardiomyopathy, 15 segments show midmyocardial septal LGE. Midmyocardial fibrosis was also found in 9 patients with hypertrophic cardiomyopathy and 70 segments showed circular/diffuse LGE in amyloidosis patients (Table 1).

### 3.2 Global Circumferential Strain

Among all patients (IHD and IHD+), individual GCS ranged widely between −3.5% to −19.8%. IHD+ patients had significantly lower average GCS than IHD patients (−12.9% vs. −16%, p < 0.05; ICC 0.898, 95%CI: 0.832-0.941). The lowest GCS was measured in patients with cardiac amyloidosis (average GCS −9.7%, range from −3.5% to −13.9%). Average GCS was higher in women than in men (−15.4% vs. −14%, p < 0.05).

### 3.3 Segmental Circumferential Strain

#### 3.3.1 Segmental Circumferential Strain in IHD and IHD+:

In IHD, average SCS was −17.4% (range −11.5 % to −22.8%) in segments without ischemic LGE and −5.9% (range 2.3 % to −7.3 %) for segments with ischemic LGE; inter-reader agreement was very good (ICC 0.881, 95% CI: 0.823-0.932). In IHD+, average SCS in segments without LGE was −16.6% (range −6.5 % to −19.1%). In myocardial segments with ischemic LGE SCS was −5.4% (range 0.4 % to −9.1 %) and in segments with non-ischemic LGE −11.8% (range −2.4% to −21.3%). Inter-reader agreement was very good (ICC 0.852, 95% CI: 0.794-0.917).

#### 3.3.2 Infarct Detection with GCS_median_ Percentage Plots in IHD and IHD+:

**Figure 3:**
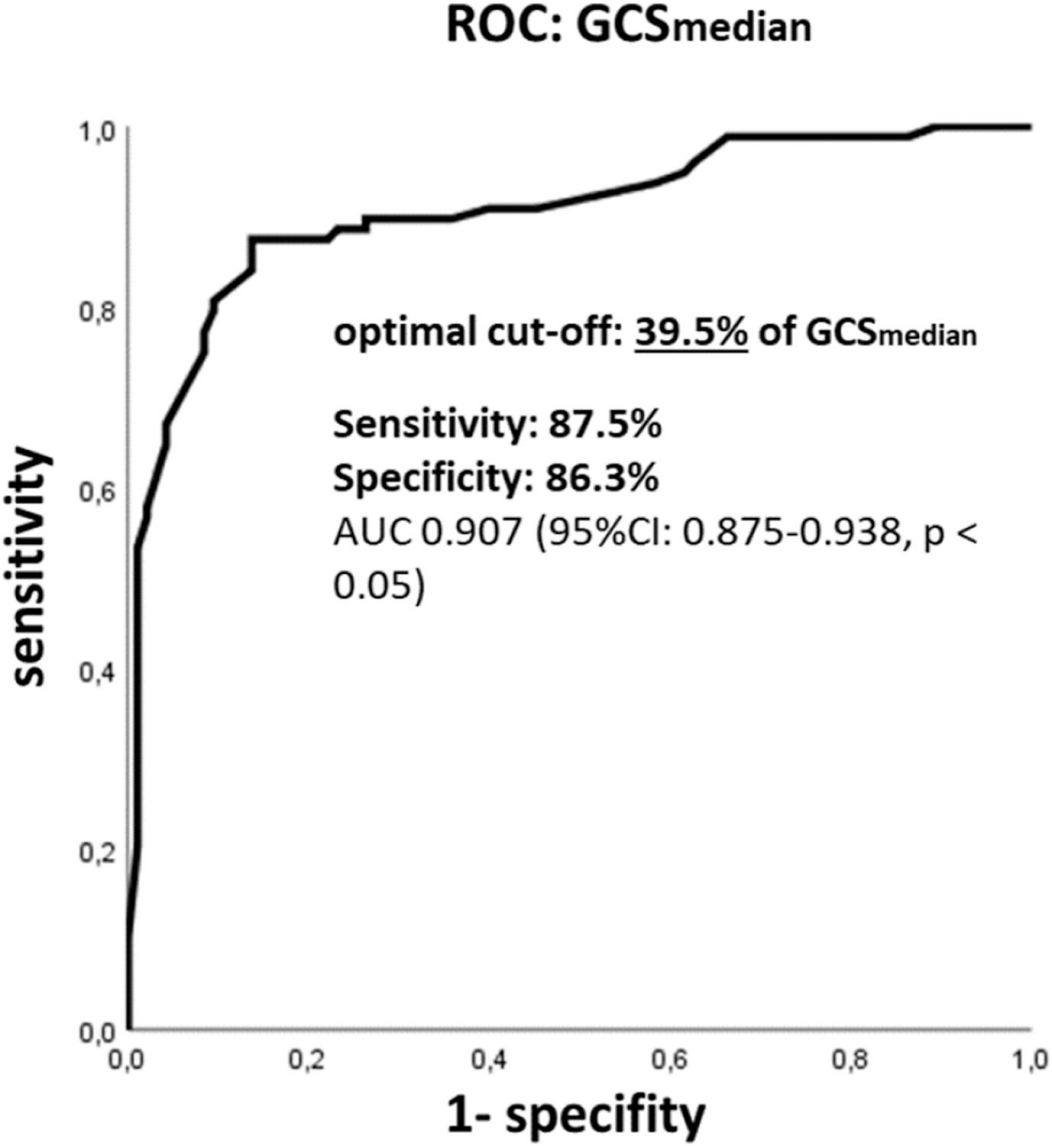
ROC curve for distinguishing infarcted and non-infarcted myocardium based on percentage values of GCS_median_. In segments with values below 39.5% patient-specific median GCS (GCS_median_), ischemic scar can be assumed with 87.5 % sensitivity and 86.3% specificity (AUC 0.907 [95% CI: 0.875 – 0.938, p < 0.05], Youden`s index 0.74). Infarcted segments in LGE short axis stacks served as gold standard. AUC- Area Under The Curve; GCS- Global Circumferential Strain; GCS_median_- Patient-Specific Median Global Circumferential Strain; LGE- Late Gadolinium Enhancement; ROC- Receiver Operating Characteristics

**Figure 4:**
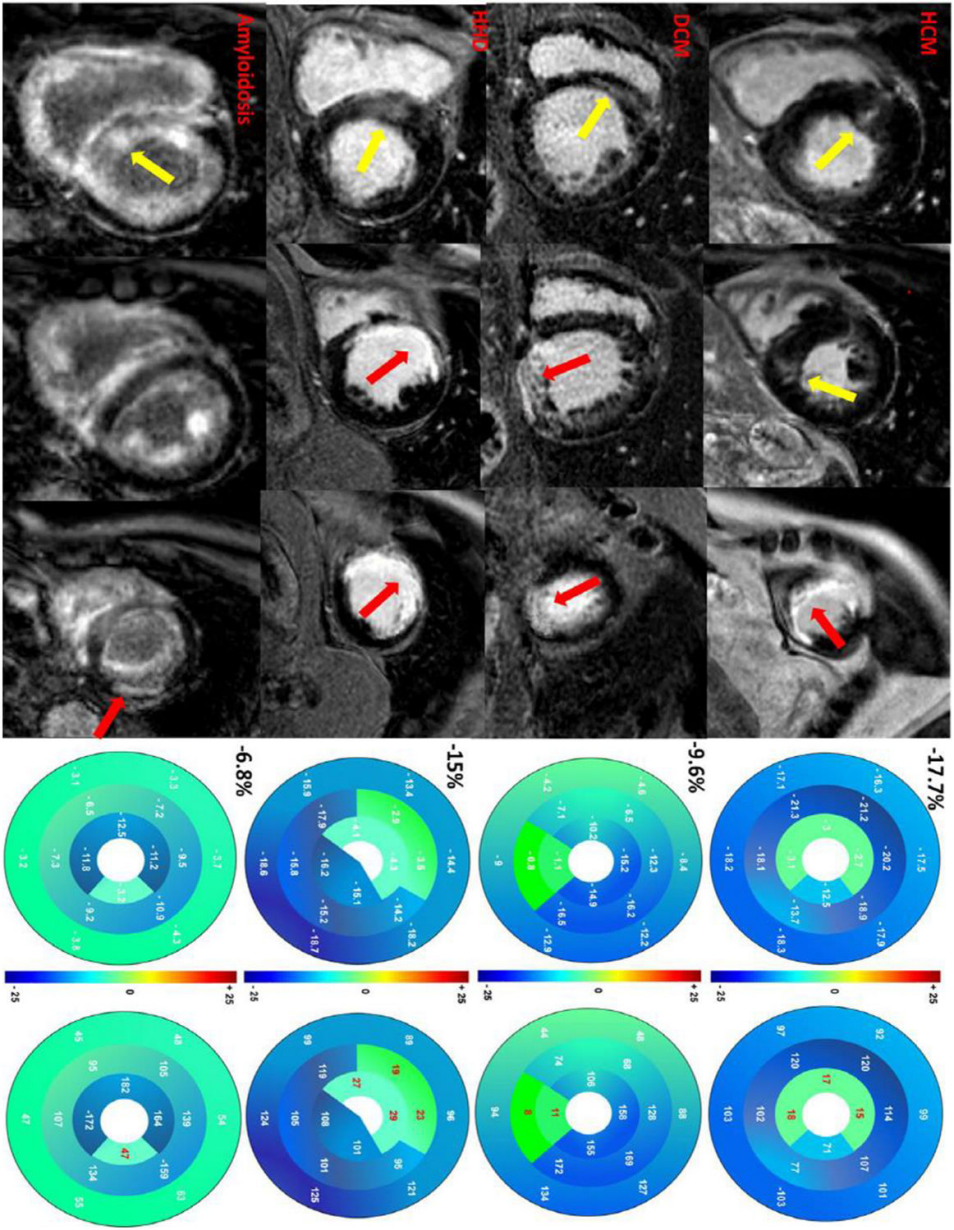
Influence of ischemic scar on segmental strain in IHD+ patients. Hypertrophic Cardiomyopathy (HCM): Diffuse subtle midmyocardial LGE anteroseptal basal and inferoseptal midventricular (yellow arrows) in a 53-year-old female patient with hypertrophic cardiomyopathy; furthermore, transmural infarction in AHA segment 13-15 can be seen. GCS_median_ is −17.7% and infarcted segments show values below 40% GCS_median_ (15%, 17% and 18%, respectively). Dilatative Cardiomyopathy (DCM): 65-year-old male patient with left ventricular dilatation, reduced LV- EF (37%), midmyocardial septal LGE in basal section (yellow arrow) and transmural infarction in segment 10 and 15. GCS_median_ is −9.6% and infarcted segments have a value below 40% GCS_median_ (11% and 8%, respectively). Hypertensive Heart Disease (HHD): 55-year-old male patient with concentric left ventricular hypertrophy, diffuse LGE anteroseptal basal (yellow arrow) and large ischemic scar in segment 7,8,13 and 14. Scar tissue displays GCS_median_ percentage values of 23%, 19%, 29 and 27%, respectively. Amyloidosis: 62-year-old male patient with amyloidosis (diffuse LGE, yellow arrow), very low GCS_median_ (−6.8%) and small scar inferior lateral (red arrow) with segment GCS_median_ percentage value of 47%, above the proposed threshold of 40% GCS_median_. AHA- American Heart Association; GCS- Global Circumferential Strain; GCS_median_- Patient-Specific Median Global Circumferential Strain; LGE- Late Gadolinium Enhancement; LV-EF- Left Ventricular Ejection Fraction

Analysis of percentage plots of GCS_median_ calculated for every patient (IHD and IHD+) revealed a cut-off value of 39.5% below the presence of ischemic LGE in a segment had a sensitivity of 87.5% and specificity of 86.3% (AUC 0.907, 95% CI 0.875-0.938, p < 0.05; Figure 3, 4).

In some IHD+ patients with GCS_median_ below - 7% (3 patients; 2 patients with amyloidosis and one with severe LV dilatation), 8 infarcted segments showed values above the proposed threshold of 40% GCS_median_ (Figure 4). Furthermore, 6 viable subendocardial scars had a strain value above the 40% GCS_median_ threshold, however, all those segments could be found in patients already diagnosed with at least one segment below the threshold. Due to exclusion of segment 17 from GCS calculation, 9 patients with infarction of the apex could not be diagnosed. Seven segments met the GCS_median_ criteria of ischemic LGE without displaying scar tissue in LGE images; further analysis revealed that strain impairment in 4 of the 7 segments were due to a LV diverticulum, clearly visible in short axis cine and 3 segments had akinesia in cine imaging without wall thinning and represented most probably myocardial hibernation. Lastly, local artifacts in cine images lead to low values in 10 segments suggesting ischemic scar but without corresponding LGE or wall motion abnormality.

## 4. Discussion

This study investigated a patient-specific approach of using CMR feature tracking for native scar detection in a heterogenous population with patients with chronic ischemic as well as non-ischemic heart disease. A threshold below 40% patient-specific median global circumferential strain (GCS_median_) indicated ischemic scar tissue in a myocardial segment with a sensitivity of 88% and a specificity of 86%. Derived from routinely acquired native cine images, CMR feature tracking displays global and segmental myocardial deformation [16]. Clinically first introduced in echocardiography, this method is also increasingly used in clinical CMR since it provides additional information from cine images beyond visual wall motion evaluation [17]. Based on the premise that most LV myocardial fibers are circumferentially orientated and thus contribute to circumferential strain, recently performed studies confirmed that local tissue destruction in ischemic scars leads to significant local circumferential strain impairment in contrast to adjacent remote tissue, allowing to distinguish remote and infarcted myocardium [5,6,18]. Hence, this technique might improve the validity of native CMR exams in patients with ischemic heart disease and contraindication to Gadolinium. However, myocardial strain can be influenced by various conditions, not only by myocardial infarction, but also by inflammatory changes, cardiomyopathies, cardiac infiltrative disease or states like hibernating myocardium, reducing specificity of segmental circumferential strain regarding scar detection in the broad population [19-21]. Therefore, we investigated a patient-specific approach in patients with chronic ischemic (IHD) and concomitant ischemic and non-ischemic heart disease (IHD+) with the aim of finding a threshold based on individual strain values, below which ischemic scars can be suspected with high probability. To our knowledge, there are no published studies yet regarding patient-specific usage of CMR feature tracking for scar detection. Analyzing the entire study cohort, global circumferential strain values ranged widely between pathologically low values (−3.5%, patient with amyloidosis and infarction) and normal GCS (−19.8%, patient with small ischemic scar) [16] with average GCS being lower in IHD+ than IHD. Interestingly, average segmental strain values in infarcted segments were similarly low in both subgroups. Non-ischemic LGE in IHD+, on the other hand, showed less severe impairment of local circumferential strain compared to segments affected with ischemic scars. This can be explained by the degree of LV wall damage caused by non-viable, ischemic scars in contrast to non-ischemic LGE with mostly preserved wall integrity. Using patient-specific median GCS (GCS_median_) - which is more robust to outliers than mean GCS - and segmental circumferential strain values, GCS_median_ percentage polar plots could be easily generated. Segment-wise correlation of the resulting polar plot values with LGE images revealed that a threshold below 40% GCS_median_ can identify the presence of ischemic scar with 88% sensitivity and 82% specificity in a heterogenous patient group with various cardiac conditions. Few infarcted segments showed values above the proposed threshold, most of them in patients with severely reduced GCS (below −7% GCS), e.g. in amyloidosis patients. In fact, identifying ischemic scars in amyloidosis with LGE imaging can be problematic as well due to often extensive diffuse LGE that might cover additional ischemic scars. Also, some viable infarcts caused false negative results with values above the proposed threshold. Viable scars might be difficult to diagnose even in LGE due to similar signal intensities between scar and adjacent myocardium and only subtle wall motion disturbance [22]. A possible approach to solve this problem could be additional acquisition and evaluation of T1 mapping with presumably higher local values in infarcted tissue compared to adjacent remote myocardium [23,24]. We noticed only few false positive results, most of them due to local artifacts. Due to a technical limitation, infarction of the apex (AHA segment 17) could not be calculated in SCS, however, infarction of the apex is often apparent in cine long axes as an apical akinesia or dyskinesia. Since only one software was used to derive global and segmental circumferential strain, further studies are required to prove if the 40% GCS_median_ threshold is also applicable for data calculated with other commercially available CMR feature tracking software. Cine images are often performed before contrast application; therefore, the proposed feature tracking method could be easily applied in a prospective clinical setting in patients with known or suspected ischemic heart disease. This method might reduce gadolinium application in future CMR exams and could improve diagnostic power of native CMR exams in patients who decline contrast or have contraindications for gadolinium.

## 5. Conclusion

A threshold below 40% patient-specific median global circumferential strain (GCS_median_) indicates chronic scars in myocardial segments with a sensitivity of 88% and a specificity of 86%. Since alternatives for detecting scar tissue from native CMR exams are limited, this patient-specific cine-based method could help to improve diagnostic power of native CMR exams in patients with contraindication to Gadolinium. However, in patients with severely reduced GCS, this method might be not sensitive enough to detect ischemic scars and additional use of tissue characterization techniques should be considered.

## Figures and Tables

**Table 1:** Patient characteristics of IHD and IHD+. BSA- Body Surface Area; BMI- Body Mass Index; GCS/SCS- Global/Segmental Circumferential Strain; LAD- Left Anterior Descending Coronary Artery; LCX- Left Circumflex Artery; LGE- Late Gadolinium Enhancement; LV-EDV- Left Ventricular End-Diastolic Volume; LV-EF- Left Ventricular Ejection Fraction; LV-SV- Left Ventricular Stroke Volume; RCA- Right Coronary Artery

	IHD n = 65	IHD+ n = 89	p- value
Demographics			
Female/male	24/41	42/47	
Age (years)	62 ± 9	60 ± 12	0.3
BSA (m2)	1.9 ± 0.4	1.9 ± 0.7	0.8
BMI	27 ± 6	26 ± 4	0.5
LV morphology			
LV-EDV [ml]	174 ± 22	185 ± 28	<0.05
LV-SV [ml]	93 ± 12	81 ± 15	<0.05
LV-EF [%]	53 ± 4	44 ± 10	<0.05
LV ischemic LGE (segments)	210	251	0.4
LAD	102	98	0.7
RCA	68	75	0.6
LCX	40	78	0.1
LV non-ischemic LGE (segments)			
	0	101	
epicardial		4	
midmyocardial		27	
circular/diffuse		70	
CMR LV strain			
Average GCS (%; range)	−16 ± 4 (−6.6 to −19.8)	−12.9 ± 5 (−3.5 to −17.7)	<0.05
Average SCS infarct (%; range)	−5.9 ± 3 (2.3 to −7.3)	−5.4 ± 2 (0.4 to −9.1)	0.5
Average SCS non-ischemic (%; range)	-	−11.8 ± 5 (−2.4 to −21.3)	
Average SCS remote (%; range)	−17.4 ± 5 (−11.5 to −22.8)	−16.6 ± 4 (−6.5 to −19.1)	0.2

## List of Abbreviations:

AHA: American Heart Association
AUC: Area Under The Curve
CMP: Cardiomyopathy
CMR: Cardiac Magnetic Resonance
GCS: Global Circumferential Strain
GCS_median_: Patient-Specific Median Global Circumferential Strain
HHD: Hypertensive Heart Disease
ICC: Intraclass Correlation Coefficient
IHD/IHD+: Chronic Ischemic Heart Disease/ Chronic Ischemic Heart Disease and Concomitant Non-Ischemic Heart Disease
LGE: Late Gadolinium Enhancement
LV: Left Ventricle/Left-Ventricular
LV-EDV: Left Ventricular End-Diastolic Volume
LV-EF: Left Ventricular Ejection Fraction
ROC: Receiver Operating Characteristics
SCS: Segmental Circumferential Strain
SSFP: Steady- State Free Precession
T: Tesla
